# Review of "Contemporary IMRT: Developing Physics and Clinical Implementation" by Steve Webb

**DOI:** 10.1186/1475-925X-4-20

**Published:** 2005-03-21

**Authors:** Vasos-Peter John Panagiotopoulos

**Affiliations:** 1 Marions Panyaught Consultancy of Samani International Enterprises, New York City, NY, USA

## 

IMRT is Intensity Modulated Radiation Therapy: Irradiating from several directions repeatedly targets a tumor, but less its surroundings, due to differing projections; yet minimising such collateral damage may be cost-controversial. Beams are *modulated *by retarding compensators and perimeter-blocking collimators. Radiotherapy retards mitosis, especially G2, but responce is tissue-specific, hampered by the likes of tumor hypoxia. X-rays result when an electron beam hits a target, but in therapy, electrons are previously electromagnetically accelerated so resulting X-rays are quite stronger. Despite improved calculation and control, nonintegration remains: Technology mismanagement frequently occurs when rogue specialties treat others as superfluous; yet the more technical and nimble a field, the greater need for cross-disciplinary clarity and integration (eg p. 379). Tradeoffs between nimbleness and safety are political moving targets. Avowedly the least mathematical, but also least focused (in any order, p.xv), of four, this tome surveys developments, more like a managerial update. Curiously the author spawned separate titles instead of revising, expanding editions: Multibook fatigue has lapsed author into shamanistic jargon and acronyms; yet such superfluous obfuscation bears significant blame for modern medical errors. The final sixth of this tome is a rich collection of references. This, or any precursor, nicely rounds off a two semester radiological engineering course with Cember's health physics[1], Faiz Khan's radiotherapy[2], Kak & Slaney's tomography[3], and Pham & Dimov's rapid manufacturing [4] texts, preceeded by thorough reexcercising of Schaum's laGrangian mechanics [5].

In the second chapter on rotation IMRT and tomotherapy, pseudo structures flagged for dose minimisation are especially interesting. The third chapter discusses multileaf collimators (MLC) and sequencers (leaf moving algorithms, aka, *curiously*, interpreters) in terms of leakage, scattering, rounding, edge penumbra and speed considerations. Radiation might be misdirected while leaves are moving and they don't move quite instantaneously, requiring Leaf Motion Calculators (*shamanistically, not *sequencers!). The fourth chapter discusses non-MLC techniques: *Accuray.com *CyberKnife (accelerators, not Gamma Knife cobalt) with real-time imaging, jaws/mask technique and variable-aperture collimators. The fifth chapter cites specific anatomical tumors and attendant clinical evidence: the author laments "evidence-based medicine" circuitously implies not being accepted until one has been accepted enough to have sufficient clinical data; Perhaps physicists disempower trials specialists. Integrated systems might compensate, real-time, for detected radiobiological characteristics like hypoxia. Furthest clinical results are Sloan Kettering's prostate (rectal toxicity declines) and Marsden's (author) breast.

3-dimensional planning spans book's last half, discussing margin definition, distortion correction, contrast agents, fluence smoothing, patient motion (esp respiration, modelled as cosine to power of assymetry) and surface marker gating, and movement correction. Tracking patient motion is preferrable over immobilisation techniques resembling medieval torture. Image importing planning softwares include Nomos *Corvus*, Nucletron *Plato*, Nordian MDS *Helax *TMS, Philips ADAC *Pinnacle*, Varian *CadPlan Helios *& CMS *Focus*. Inverse planning minimises discrepancy from desired spatial dose, constrained by dose (power-law biological cost) to non-targets and then projects beams backwards from each direction. Various optimisation methods are discussed, quite improved from 1960s confinement to linear programming; However, one must avoid that optimiser scourge, local minima. Monte Carlo techniques (MCDOSE, DOSXYZ, MCNP, ETRAN, EGS4; accumulating random beam behaviors defined by Boolean rules instead of differential equations) allow for simulation of geometries not easily described mathematically, but are slow and have noise convergeance errors.

Since Cyberknife robot "chases the moving patient" (p. 164), one hopes for more engineering, integrating "planning" with therapy, instead of "batch" methods, which the author prefers, like older automobiles, in his 2001 text, because they do "not remove the human from decision making". Engineers would prototype in *MathWorks.Com MatLab*(p. 47), but would consider it too slow for "*production*". Planning originated with slower computers (before MLC sequencers, p. 42) like Harvard JCRT (Bjarngard, Kijewski, Siddon) [6], Wachsmann's 1959 pendulum and rotation "Moving Field Radiation Therapy" [7] and van de Goijn's 1970 3D planning EXTDOS; yet Shirato & Sawada (pp. 335–341) develop real-time planning integration and Tubingen (p. 295) integrates MLC constraints, sequencers and planning. Given mathematical computation now so competent at scavenging useful information from what was previously considered noise (eg Barbour, *Nirx.Net*), perhaps one might exploit treatment radiation for some imaging, reducing total exposure, and seek further integration synergies.

## Competing interests

Reviewer holds restricted securities in a developer of a through-the-sputtered-target, minimal-Bremsstrahlung, relativistically-collimated miniaturised X-Ray tube.
